# Cincinnati Schools of Medicine

**Published:** 1855-09

**Authors:** 


### CINCINNATI SCHOOLS OF MEDICINE.

There has been a project on foot to consolidate the Ohio Medical College and the Miami School, in that city; but it appears that there has been some difficulty in arranging the terms of union.— The old School proposed to submit the claims of the Faculties of both schools to public trial, and those found most worthy should be selected by the Trustees of the Medical College of Ohio.

If this trial take place before medical men, and the matter submitted to their decision, we would find no objection to it, but would approve of it. But if to a miscellaneous class of non-medical men the whole scheme would, in our opinion, fail of ultimate success. The proposition was *fair* enough on the part of that College, but was declined by the Miami College for reasons not stated.

We have ever believed that, considering the fact that there are two other well established and highly reputable schools in Ohio, there was no necessity for more than one in Cincinnati. That institution in possession of the best buildings, and the most appliances for teaching should be retained. If we are not misinformed, the State, at one period, made a bestowment of means in aid of the Ohio Medical College, and if so, the fund so appropriated should not be lost, but we trust that the Trustees will be able to agree upon a Faculty composed of men of learning, science, and of stern integrity. As we have said heretofore, an institution can not stand against the blighting influences of even one man connected with it, who is lost to all principle, and looking to nothing but the gratification of his own mad, uncurbed ambition, to gratify which, "the end is made to justify the means." Conciliation toward such may do, where there are a few moral obliquities, but where the vices predominate, then a stern inflexible decision should characterize the acts of those who love justice and right, above all other considerations.

There are worthy, talented, and learned men in both of the institutions in Cincinnati, and we are sure an able Faculty can be formed from the material in that city, whether in the schools or out of them. Prof. Armor, our old friend and colleague, is Dean of the Faculty of the Ohio Medical College, and is an accomplished teacher, as well as a scientific man. A Faculty made up of such men, cannot but give satisfaction and contribute largely to the advancement of medical science in that region.

